# Supplementary material for: Three-dimensional inversion of gravity data using implicit neural representations and scientific machine learning
Source: Sci Rep. 2026 Jun 4;16:25630. doi: 10.1038/s41598-026-55960-5 (PMC13478334; doi:10.1038/s41598-026-55960-5)
Supplement: Supplementary file 1 [file 41598_2026_55960_MOESM1_ESM.pdf]

Supplementary material

Three-dimensional inversion of gravity data using implicit neural representations and scientific machine learning

Pankaj K Mishra [1*], **Sanni Laaksonen** [1], **Jochen Kamm** [1], **Anand Singh** [2]

[1]Geological Survey of Finland, Vuorimiehentie 5, Espoo, Finland
[2]Indian Institute of Technology Bombay, Mumbai, India
*Corresponding author: pankaj.mishra@gtk.fi

Noise Sensitivity of the INR Inversion

To evaluate the robustness of the INR-based gravity inversion to data contamination, we carried out a controlled noise-sensitivity study using the same synthetic block-model setup as in the previous experiments. The computational domain spans $1000 \times 1000 \times 500$ m and is discretized with 50 m cells. The true density model consists of a staircase-shaped body with density contrast $400\ \mathrm{kg/m^3}$ embedded in a zero-background medium. Gravity data are computed on a regular surface grid using the same forward operator as in the preceding tests.

To isolate the effect of noise alone, the inversion configuration was kept fixed across all experiments: the INR architecture, positional encoding (two frequency bands), optimiser (Adam), learning rate, training schedule, and early-stopping criterion were identical. Only the noise model and noise amplitude were varied. The stopping rule follows a discrepancy principle, targeting a whitened data misfit $\chi^2 = \mathrm{mean}[(r/\sigma)^2] \approx 1$.

Three noise classes were considered. The first is Gaussian noise, which serves as the reference case. The second is spatially correlated noise, introduced to mimic survey and processing effects such as drift, leveling errors, or imperfect background removal. The third is sparse outlier noise, representing localized corrupted observations. Each noise type was tested at amplitudes of 2%, 5%, and 10% of the standard deviation of the noise-free gravity response, extending the 1% baseline used in earlier experiments.

Figure 1 summarises the recovered models. The y–z sections show that the INR inversion preserves the main geometry and location of the target body across all nine noise conditions. Variations appear primarily in amplitude and boundary sharpness as noise increases. The Gaussian and correlated noise cases produce very similar reconstructions, indicating that moderate spatial correlation in the data errors does not significantly affect the recovered structure. The outlier-noise cases show more visible degradation, but the anomaly remains clearly identifiable.

The convergence histories in Figure 2 provide additional insight into optimisation behaviour. For Gaussian and correlated noise, the weighted misfit χ^2 decreases rapidly and approaches the target noise level in a stable manner. In contrast, the outlier-noise cases exhibit slower convergence and oscillatory behaviour. This is consistent with the use of a squared-error loss, which is known to be sensitive to outliers. Despite this, the inversion remains stable and converges to models that reproduce the dominant structure of the anomaly.

Quantitative metrics are consistent with the visual interpretation. Across the Gaussian and correlated cases, the final model errors vary only moderately with increasing noise amplitude. Even at the 10% level, the recovered structures remain comparable in geometry to those obtained at lower noise levels. The outlier cases show higher model error and misfit variability, but the degradation is gradual rather than abrupt.

These results indicate that the INR inversion is robust across a range of realistic noise conditions. Performance is strongest for Gaussian and correlated noise, while sparse outliers produce the largest degradation, as expected for an L_2 data-misfit formulation. The method degrades progressively rather than failing, and continues to recover the principal subsurface structure even under substantially noisier data. This behaviour suggests that the INR framework does not rely on a narrowly idealised noise model and remains applicable under conditions relevant to practical gravity inversion.

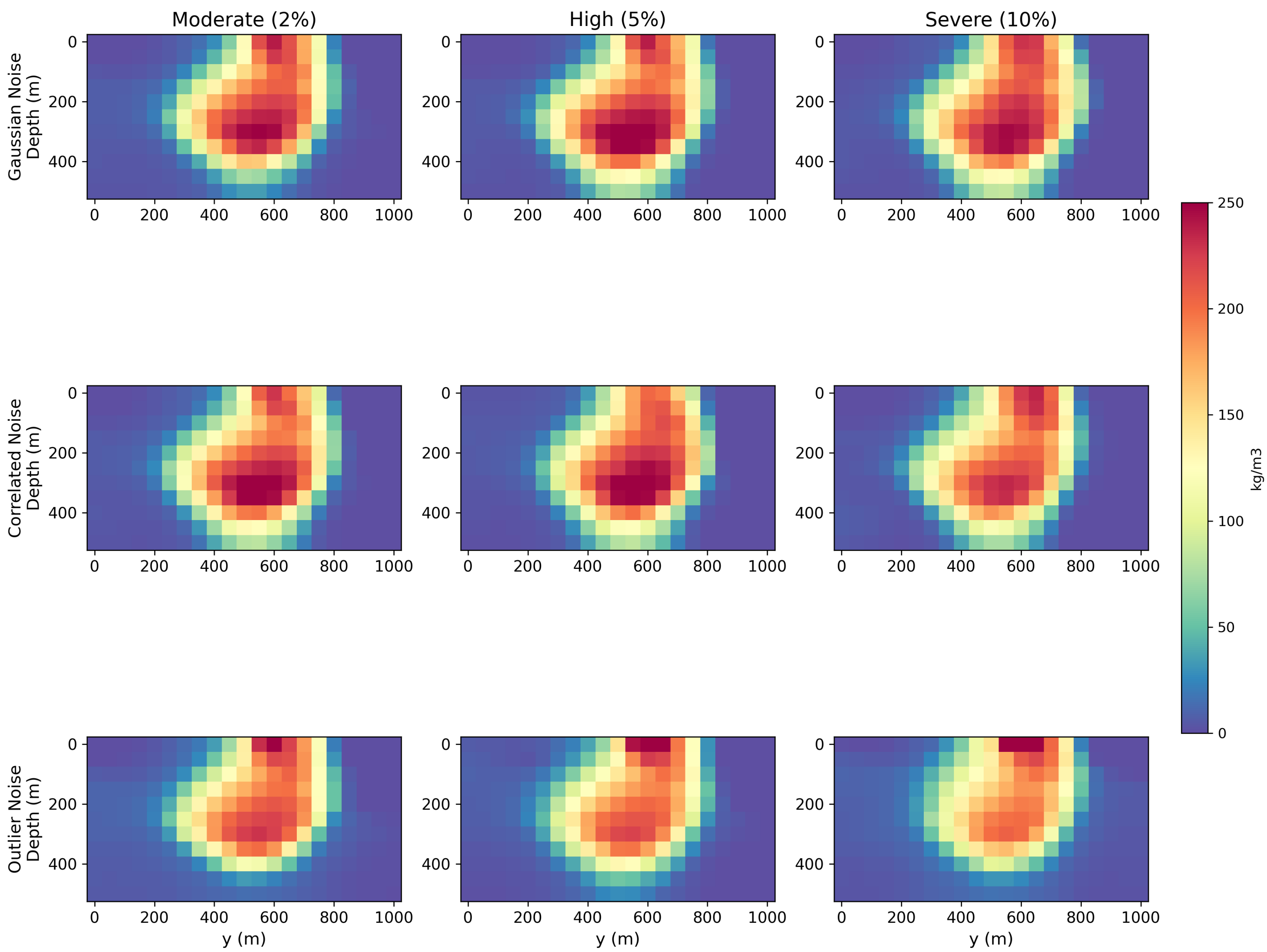

Figure 1: Recovered y–z sections from the INR inversion for three noise classes (Gaussian, correlated, and outlier noise) and three noise levels (2%, 5%, and 10%). The main geometry and location of the staircase-shaped target are preserved across all tested conditions. Gaussian and correlated noise yield very similar reconstructions, whereas outlier contamination produces somewhat stronger degradation in amplitude and boundary sharpness.

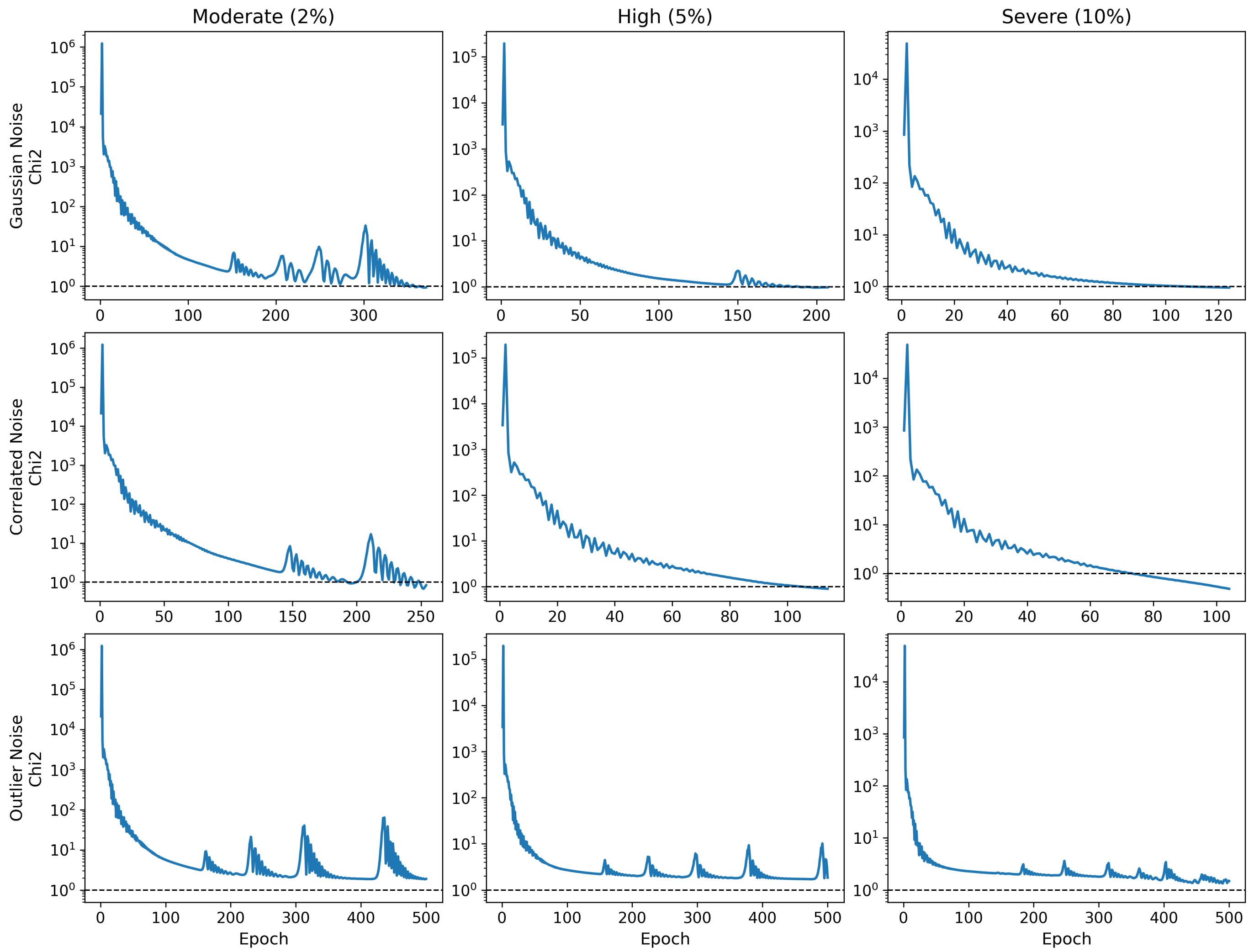

Figure 2: Convergence histories for the noise-sensitivity study, shown in terms of the weighted data misfit, χ^2, for the same nine cases as in Figure 1. The dashed horizontal line marks the target noise level. Gaussian and correlated noise cases converge stably toward the target, while outlier-noise cases show slower and more oscillatory behaviour, consistent with the sensitivity of the squared-error loss to isolated bad observations.
